# AARDVARK: an automated reversion detector for variants affecting resistance kinetics

**DOI:** 10.1093/bioinformatics/btad509

**Published:** 2023-08-16

**Authors:** Thaidy Moreno, Joaquin Magana, David A Quigley

**Affiliations:** Department of Urology, UCSF, San Francisco, CA 94158, United States; Graduate Program in Biological & Medical Informatics, UCSF, San Francisco, CA 94158, United States; Department of Urology, UCSF, San Francisco, CA 94158, United States; Helen Diller Family Comprehensive Cancer Center, UCSF, San Francisco, CA 94158, United States; Department of Epidemiology & Biostatistics, UCSF, San Francisco, CA 94158, United States

## Abstract

**Summary:**

Resistance to two classes of FDA-approved therapies that target DNA repair-deficient tumors is caused by mutations that restore the tumor cell's DNA repair function. Identifying these “reversion” mutations currently requires manual annotation of patient tumor sequence data. Here we present AARDVARK, an R package that automatically identifies reversion mutations from DNA sequence data.

**Availability and implementation:**

AARDVARK is implemented in *R* (≥3.5). It is available on GitHub at https://github.com/davidquigley/aardvark. It is licensed under the MIT license.

## 1 Introduction

**Figure 1.**
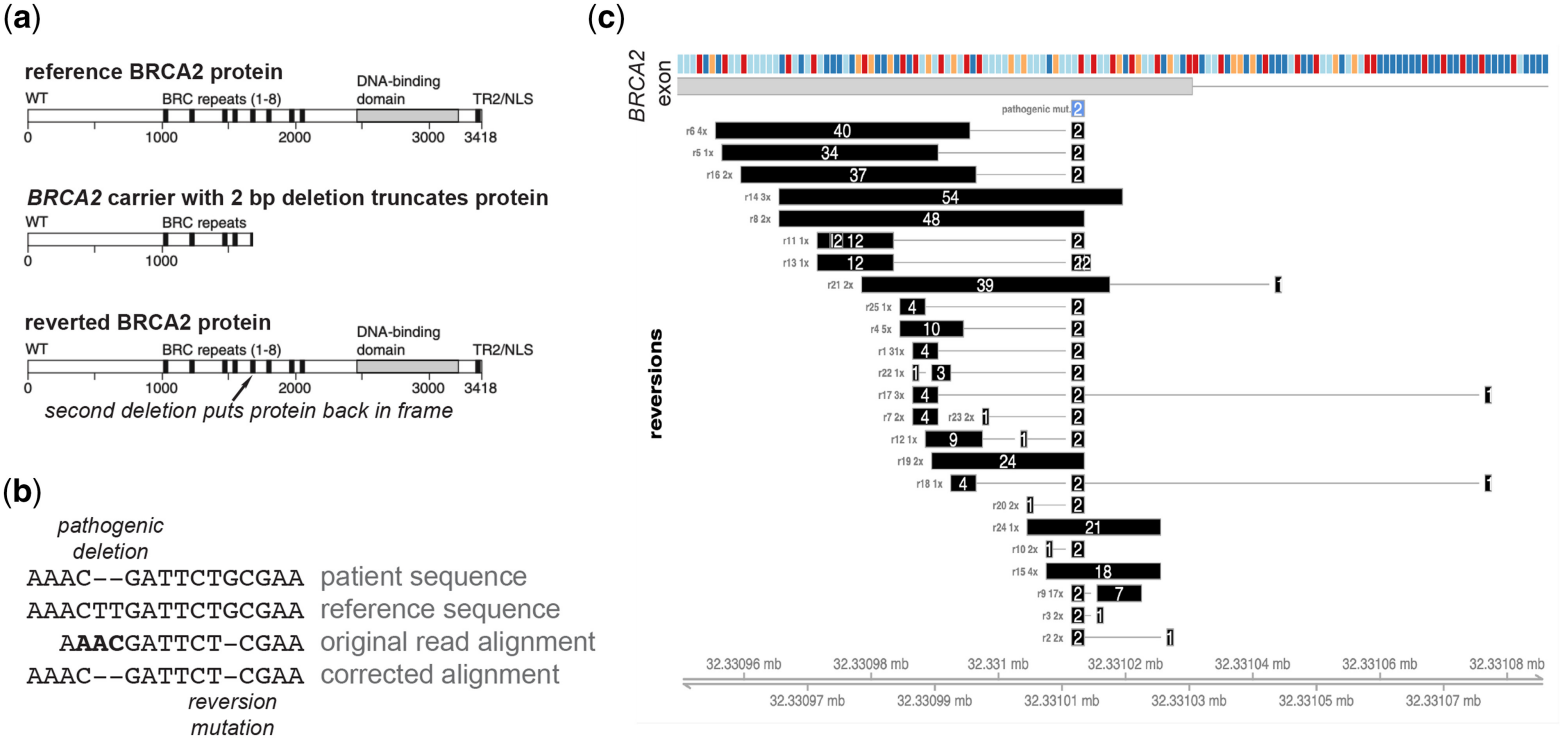
AARDVARK can identify and display reversion mutations. (a) A two-nucleotide deletion terminates the BRCA2 protein before the essential nuclear localization signal (NLS), inactivating it. An additional four nucleotide deletion can restore the *BRCA2* reading frame. (b) A germline two nucleotide deletion in the coding region of *BRCA2* compared to the reference (deletions shown as dashes), produces an inactivated protein. The third line shows the left-most 14 nucleotides of a 150-nucleotide read. The original suboptimal alignment assigns the germline deletion as a mismatch (bold text) and single base deletion, with a CIGAR string of 10M1D140M, and misses the reversion. AARDVARK corrects this alignment to 4M2D6M1D140M, and identifies a total of three bases were deleted, restoring the *BRCA2* reading frame. (c) AARDVARK applied to data from Quigley *et al.* (2017) identified and plotted 25 distinct reversion mutations identified in the same patient by exome sequencing of circulating cell-free tumor DNA.

Inherited mutations that inactivate a protein essential for homologous recombination repair of double strand DNA breaks elevate a person's risk of developing breast, ovarian, and prostate cancer (Lord and Ashworth 2016). These pathogenic mutations are rare in the general population but are clonally present in every cell of individuals who carry pathogenic *BRCA1/2* alleles in their germline. Tumors that develop in people born with a mutated copy of the *BRCA1* or *BRCA2* gene, which are both essential for homologous recombination repair, frequently lose the functional *BRCA1/2* allele. These homologous recombination deficient tumors are vulnerable to poly(adenosine diphosphate–ribose) polymerase inhibitors (PARPi) and platinum therapies through a synthetic lethality mechanism (de Bono *et al.* 2020; Farmer *et al.* 2005; Fong *et al.* 2009; Lord and Ashworth 2016). Despite the benefit offered by these drugs, acquired therapy resistance is a major clinical challenge. Selective pressure from platinum or PARPi favors the expansion of rare alleles harboring reversion mutations that re-activate the broken *BRCA1/2* gene, resulting in therapy resistance (Edwards *et al.* 2008, Quigley *et al.* 2017, Christie *et al.* 2017, Loehr *et al.* 2023) (Fig. 1a). Reversion mutations arise many times within the same tumor mass, with numerous individual reversion mutations identified at low variant allele frequency (1–5%). Because the individual mutations may be supported by low numbers of sequencing reads and because sequence alignment solutions that do not appear to produce a reversion mutation may be favored by the default parameter settings for DNA alignment software, reversion mutations are usually not identified with high confidence by general purpose variant identification tools. Manual annotation of DNA sequences to identify reversion mutations requires laborious curation and genetics expertise and can miss complex or unusual reversions. To overcome these challenges, we developed a computational approach that identifies reversion mutations effectively and without human intervention. This software tool is useful for detecting and monitoring therapy resistance and could also be applied in clinical reporting pipelines.

## 2 How AARDVARK automatically identifies reversion mutations

We developed AARDVARK (An Automated Reversion Detector for Variants Affecting Resistance Kinetics), an R package that identifies reversion mutations in DNA sequence data. AARDVARK produces a summary of all alleles where a candidate pathogenic mutation is identified and reports the reads supporting those alleles. DNA sequencing reads harboring reversion mutations are enriched for situations where genome alignment scoring does not produce an optimal result. Alignment tools such as *bwa* (Li and Durbin 2009) apply scoring rules that give good results in most cases but can produce suboptimal alignments around pathogenic mutations. By concentrating on genomic regions near pathogenic variants, the only location where reversion alleles can be found, AARDVARK can perform focused analysis that would be too computationally expensive to perform genome-wide. Any mutation downstream of an introduced stop gain would not produce a reversion, because the polymerase will stop at the novel stop codon. Reversion mutations that are small insertions or deletions tend to be <200 nt from the pathogenic mutation (Quigley *et al.* 2017). AARDVARK exploits the prior knowledge that a pathogenic mutation is present and that rare variants that restore a gene's reading frame could be enriched in the immediate vicinity of the pathogenic mutation. This additional information can identify improved alignments using the prior knowledge that a pathogenic mutation exists, instead of relying on alignment rules that are broadly appropriate for unbiased genome sequence alignment.

AARDVARK can correct several problems that impact identification and interpretation of reversion mutations. Reversion mutations can take the form of large deletions that in principle could be detected by structural variant callers, but which are in practice not reported due to their low variant allele frequency. To identify these alterations, AARDVARK first checks whether the alignment is soft-clipped at the 5-prime or 3-prime end. If so, AARDVARK performs Smith-Waterman alignment of the soft-clipped section of the read within a user-defined alignment window. If a high-quality match is identified for the soft-clipped region (defined as a continuous segment of at least a user-defined minimum number of perfectly matched nucleotides), the soft-clipped region is converted to a matched region.

Another case where AARDVARK improves alignments occur when the leading or trailing edge of a DNA read overlaps a pathogenic deletion (Fig. 1b). After checking for soft-clipped ends, the matched portion of the read is locally realigned. If the 5-prime or 3-prime end of the matched region overlaps the patient's pathogenic mutation and contains mismatches, AARDVARK attempts local realignment using a genome rewritten with the patient's pathogenic mutation. For this situation, AARDVARK uses alignment scoring parameters that do not heavily penalize small deletions or insertions, since these are the most common form of reversion mutation. If this comparison produces an alignment with a perfect match to the personalized genome model, the new alignment is accepted.

AARDVARK also identifies cases where the nucleotides deleted in a pathogenic mutation are adjacent to DNA sequence that is identical to the deletion. One example of this situation would be a reference sequence ACGAGAT where the pathogenic mutation eliminates the first GA dinucleotide, producing AC–GAT. General purpose aligners may align this deletion over the second dinucleotide, which is genetically implausible, instead of placing it over the first dinucleotide, which matches the patient's pathogenic mutation. During local realignment, AARDVARK tests whether aligning reads to a modified reference genome rewritten to include the pathogenic variant produces a higher-quality alignment than the standard reference. If AARDVARK detects this specific situation, the alignment is rewritten to be more genetically plausible, permitting the potential identification of a reversion mutation. AARDVARK is aware that homopolymer regions are a common source of sequencing errors on the Illumina platform (Stoler and Nekrutenko 2021). Homopolymer regions are defined as adjacent repeats of the same nucleotide (defaulting to five and adjustable by the user). AARDVARK automatically identifies homopolymer regions in the alignment window and flags candidate reversion variants that overlap these regions. After all realignment steps are complete, AARDVARK creates a predicted protein translation using the gene's transcript rewritten to reflect the modified read. If the modified transcript now produces an intact protein with the same stop codon as the canonical version, the read is marked as a candidate reversion allele. Users can supply a custom transcript model for this comparison or use canonical transcripts automatically downloaded from Ensembl using the *biomaRt* package.

AARDVARK can be run within R or from a command line script. Sequencing reads for the input data typically are read from a standard BAM-formatted file. AARDVARK can be run using any genome reference. Any number of pathogenic variants can be tested from a standard Variant Call Format file. AARDVARK will generate a reversion report for each variant, and has built-in plotting functions to display the results using the Gviz package (Hahne and Ivanek 2016) (Fig. 1c).

## 3 Example application of AARDVARK to genome sequence data

To demonstrate the use of AARDVARK, we applied it to DNA sequence data generated from circulating cell-free tumor DNA in a patient with metastatic prostate cancer who had progressed on PARPi therapy (Quigley *et al.* 2017). Germline testing indicated this patient harbored a pathogenic two nucleotide deletion in the coding sequence of *BRCA2*. Reads were aligned using bwa mem version 0.7.17-r1198-dirty (Li and Durbin 2009) against reference genome NCBI GRCh38 PAR-masked with decoys hs38d1. Read duplicates were marked for filtering using *Picard* version 2.23.8 (http://broadinstitute.github.io/picard/). The BAM file was sorted and indexed using *samtools* version 1.9-93-g0ca96a4 (Li *et al.* 2009). Applying AARDVARK to re-align the DNA near this pathogenic alteration automatically identified 25 reversion alleles. AARDVARK reported the reversion alleles and the read identifiers supporting them to a text file. We then used AARDVARK to plot the reversion alleles and their frequencies from this report (Fig. 1c).

## 4 Conclusion

AARDVARK automatically analyzes genome sequence data to identify patients who have developed resistance to PARPi and platinum, two widely used classes of FDA-approved therapies. AARDVARK can be incorporated into clinical reporting of patient solid tumor or circulating tumor DNA sequencing to predict whether patients harbor resistance to PARPi or platinum therapy.

## Data Availability

The software and data underlying this article can be downloaded from the Zenodo repository at https://doi.org/10.5281/zenodo.8226395. The software described in this article is freely available at https://github.com/DavidQuigley/aardvark.
